# Emergence of Life

**DOI:** 10.3390/life1010007

**Published:** 2011-09-29

**Authors:** Marie-Paule Bassez

**Affiliations:** Département Chimie, IUT Robert Schuman, Université de Strasbourg, 72 Route du Rhin, 67400 Illkirch, France; E-Mail: marie-paule.bassez@unistra.fr

Le tout n'est pas la simple addition de toutes les parties.

(The whole is not the simple addition of all the parts.)

Indeed, even if we know that many individual components are necessary for life to exist, we do not yet know what makes life emerge. One goal of this journal *Life* is to juxtapose articles with multidisciplinary approaches and perhaps to answer in the near future this question of the emergence of life.

Different subjects and themes will be developed, starting of course with the multiple definitions of life and continuing with others such as: life diversity and universality; characteristics of living systems; thermodynamics with energy and entropy; kinetics and catalysis; water in its different physical states; circulation of sap and blood and its origin; the first blood pump and first heart; the first exchange of nutrients between cells, sap and blood; essential molecules of living systems; chirality; molecular asymmetry and its origin; formation of enantiomer excess and amplification; microscopic observations on a micrometer and sub-micrometer scales, at molecular and atomic levels; the first molecules at the origin of genetic information, viroids, circular RNA; regions of space or the area inside membranes and cells capable of initiating and maintaining life; phenomena at the origin of the emergence of life; molecules studied in the traditional field of chemistry and in the recent field of nanoscience governed by new laws; interaction between the individual molecules and components of living systems; interaction between living systems and the environment; transfer of information through generations; continuation of life from one generation to the next; prebiotic chemistry and prebiotic signatures on Earth, on Mars, on other planets; biosignatures of the first forms of life; fossils and pseudofossils dating 3.5 Ga ago and more recent ones; experimental fossilization; pluricellular eukaryotes dating 2.1 Ga ago; sudden increase in oxygen in the atmosphere around 2.0 to 2.5 Ga ago and its relation to geology; shell symmetry; aging with transformation of molecules, of their symmetry, their interactions, their exchanges…

This journal invites scientists to participate in a multidisciplinary approach toward understanding life, its origins, its evolution, its distribution from the surface of the Earth to the inside of rocks and to the extreme terrestrial and extraterrestrial physico-chemical conditions. Articles arising from the fields of chemistry, physics, biology, exobiology including the search for “analogues”, space science, geology, climatology and atmospheric sciences of the primitive Earth and of other planets, analytical chemistry, paleontology, mathematics, and arising also from other disciplines are welcome to contribute to the goal of this journal: *Life*. The assemblage of different specific fields of expertise will certainly lead to innovative ideas for the global understanding of life, its birth, its evolution, and its transfer through generations.

